# The President’s Physicians

**Published:** 1881-10

**Authors:** 


### The President's Physicians.

While the public has been commenting upon the illness and death of our late President, not a few remarks have been made concerning his much advertised doctors.

The nation mourns a loss almost irrepairable, yet it is, at least, a matter of congratulation to physicians in general, that the conduct of the medical attendants in the case has been of such a nature as to win the confidence of the entire community. While occupying such a prominent position before the public, they have been subjected to both annoyance and temptations of rather a peculiar nature. They have been overwhelmed by gratuitous advice from all sorts of people, and nostrums of various forms have been urged upon their notice, sometimes in friendly simplicity, but usually with the hope of obtaining for the remedy and its suggester an advertisement free of charge. The treatment of the distinguished patient has been sharply criticized by more than one doctor of small practice and smaller mind, as he entertained his astonished friends with an exhibition of his own wonderful insight into the President's condition; and still worse, abundant advice has been publicly offered by one or two of metropolitan notoriety who ought to have a better knowledge of the medical code of ethics.

Especially great was the temptation for the attending physicians to make themselves unnecessarily conspicuous, but though one of them has occasionally shown a fondness for the society of reporters, yet, as a rule, a most becoming dignity was observed in regard to official bulletins. From the very first, they have presented the facts of the case in a distinct, straightforward manner. The condition of their patient has been stated as nearly as possible, with mathematical accuracy, and the plan of treatment frankly and fairly given. Since the autopsy, it has been rather a matter of surprise that a man, wounded so severely, should live so long, and although the result was so much deplored, it is a source of great consolation to know that the late President received the best attention of two of the most skillful and distinguished surgeons of the land, and that their conduct has reflected considerable credit on the profession to which they belong.

